# Adapting Short-Term Mentalization-Based Therapy to ICD-11 Personality Disorder in Adolescents

**DOI:** 10.3390/children10010093

**Published:** 2023-01-02

**Authors:** Sebastian Simonsen, Emilie Hestbæk, Sophie Juul

**Affiliations:** 1 Stolpegaard Psychotherapy Centre, Mental Health Services in the Capital Region of Denmark, 2820 Gentofte, Denmark; 2 Copenhagen Trial Unit, Centre for Clinical Intervention Research, The Capital Region of Denmark, Copenhagen University Hospital–Rigshospitalet, 2820 Copenhagen, Denmark

**Keywords:** personality pathology, ICD-11 personality disorder, short-term mentalization-based therapy, adolescents, short-term psychotherapy

## Abstract

Following the introduction of the 11th revision of the International Classification of Diseases (ICD-11), adolescents can now be diagnosed with a personality disorder based on severity ranging from mild to moderate to severe. This dimensional model has potential implications for treatment, as it allows clinicians and researchers to search for effective treatments targeting adolescents at different severity levels rather than offering all patients the same treatment. In this conceptual paper, we propose that the short-term mentalization-based therapy (MBT) program, originally developed to treat adults with borderline personality disorder (BPD), has potential clinical advantages for adolescents with ICD-11 personality disorder at the mild to moderate severity level. The short-term MBT program is a 5-month structured treatment approach including individual therapy, combined psychotherapy with the individual therapist also being one of the group therapists, and closed-group therapy to enhance cohesion and a feeling of security. The purpose of this paper is to make a case for the use of this format, as opposed to the traditional long-term MBT format, for adolescents with BPD. Future research should include large-scale randomized clinical trials powered to assess patient-important outcomes.

## 1. Introduction

The 11th revision of the World Health Organization (WHO)’s International Classification of Diseases (ICD-11) represents a paradigm shift in the diagnosis of personality disorders from a categorical to a dimensional model [1]. When it comes to diagnosing children and adolescents with personality disorders, a developmentally informed position is maintained in ICD-11, as it is stated that “manifestations of personality disturbance tend to appear first in childhood, increase during adolescence […]“ [2] (p. 388). However, clinicians are simultaneously urged to exercise caution in assigning the personality disorder diagnosis to children and adolescents. The fact that clinicians can diagnose personality disorders in children and adolescents, but are advised not to, is not a major change from ICD-10 [3]. Instead, the major changes seem to lie both in the ICD-11′s capacity to differentiate different levels of personality disorder severity and in the provision of a new psychological language for describing individual differences in personality functioning applicable across a lifespan. Differentiating between mild, moderate, and severe personality disorder may cause clinicians to be less reluctant to diagnose personality disorder of, e.g., mild severity in adolescents, especially if they can refer these adolescents to relevant and effective treatments targeting aspects of self and interpersonal functioning. The change from symptom categories in ICD-10 to dimensions describing personality function (e.g., identity, affect regulation, perspective-taking, empathy, etc.) in ICD-11 is profound because it grounds personality disorders in a developmental and psychological language that assumes continuity between normality and pathology rather than relying on arbitrary cut-offs. This change will hopefully reduce the negative effects of labeling and stigmatization and make the personality disorder diagnosis better understood and more acceptable to both the afflicted person and their surroundings, including clinicians, family, and friends. By providing a language grounded in normal development, the ICD-11 diagnostic system moves towards what might be labeled academic psychology, asking questions such as: How does identity develop? How do we learn to regulate emotions and understand one’s own and other’s minds? Instead of potentially distancing ourselves from the diagnosis by using somewhat alienating labels and descriptors, such as borderline, paranoid, or dependent, we are now forced to regard social context as part of both the cause and the cure in order to help the individual with a personality disorder. For an overview of examples in language for personality disorders between ICD-10 and ICD-11, see Table 1.

A dimensional understanding grounded in developmental psychology also provides a new roadmap for intervention science aimed at alleviating suffering, because the model suggests that helping people with the disorder implies creating circumstances and processes around the person similar to those we know are important in normal development (salutogenesis) [4] We believe that the concept of mentalization has unique relevance for understanding the personality functioning described in the ICD-11 and, furthermore, that mentalization-based therapy (MBT) can be adapted to fit the needs of patients across the entire dimension of personality functioning [5]. Mentalizing is a broad term overlapping with numerous other terms within the field of social cognition. At its core, the term concerns how we make sense of ourselves and others [6]. However, providing MBT to children and adolescents suffering from personality difficulties or personality disorders may require specific modifications of the original MBT models used in adult populations. The purpose of this conceptual paper is to develop the idea of why and how the short-term MBT program [7] can be adapted to fit the needs of adolescents battling personality disorder at a mild to moderate level of severity. In terms of the ICD-11 personality disorder classification, we will not cover all diagnostic requirements but focus on severity/functioning determinants.

## 2. Mentalization and Personality Functioning

The abandonment of the categorical approach based on symptom categories to a dimensional approach based on severity is a radical shift that carries both risks and opportunities. In the context of diagnosing and treating children and adolescents, one possibility is that the ICD-11 system will be able to capture young people at an earlier stage of disorder and that clinicians then can refer them to psychological treatment with a potential preventive impact. The ICD-11 personality functioning dimension is highly influenced by and congruent with the Diagnostic and Statistical Manual of Mental Disorders (DSM-5) Alternative Model of Personality Disorders (AMPD) [8]. The AMPD defines personality functioning through two domains of self (identity and self-direction) and interpersonal capacities (empathy and intimacy). Bender and colleagues [9] based the content of the AMPD personality functioning dimension on a survey of clinician-rated measures of self and other pathology and acknowledged the concept of mentalization as central to the severity measure. We believe that the centrality of mentalization also holds true for the ICD-11 personality disorder guidelines. Understanding personality disorder severity through the lens of mentalization has important implications, as the manifestations of the disorder are developmental in origin, dynamic, and process-based rather than a reflection of static traits inherent in the individual. This helps position the problems of young people with problems of, for example, identity formation and self-worth, as contextual, and the process of enhancing mentalizing as something that is central to both the afflicted person and their surroundings [10]. Mentalization is a multidimensional concept that can be broken down into a set of polarities (implicit/explicit, self/other, affect/cognition, and internal/external), and determinants of personality disorder severity seem to fit nicely onto the mentalizing poles. As an example, consider the cognitive manifestation “accuracy of situational and interpersonal appraisals, especially under stress” [2] (p. 378). This characteristic may be viewed as a mentalizing problem of other, self, and cognitive polarity. The MBT approach would be to clearly outline situations and relationships in which this problem manifests and target increased mentalizing by interventions aimed at balancing the poles. In most cases when problems with the accuracy of situational and interpersonal appraisals are pervasive, the mentalizing therapist may also recognize a state of psychic equivalence in the person and will work hard to shift this state by focusing on process rather than content [11].

Another potentially attractive feature of the ICD-11 personality classification is the ability to differentiate the severity of disorder, which may help guide clinicians towards more tailored treatments. Thus, for example, in the case of mild personality disorder, under stress, the person would manifest with some distortions in appraisals, while distortions in appraisals would be marked in moderate personality disorder and extreme in severe cases. Although this probably resonates with most clinicians who recognize that there may be large variability in stress reactions, it is uncertain how well clinicians in clinical practice will be able to assess such differences among their patients. Even within research settings where reliable clinical measures have been developed and are used, the clinical utility and implications of differences in severity remain largely untested [12]. To the best of our knowledge, no randomized controlled trial has randomized patients with personality disorders of the same severity level into two distinctly different experimental and control groups, and based on the evidence of a strong common factor across bona fide treatments, it is not obvious that we should expect to find large effect differences across different levels of severity [13]. This knowledge gap in the implications of the ICD-11 for treatment may be even wider when it comes to children and adolescents. In this conceptual paper, we will, thus, primarily review the evidence base for MBT in children and adolescents with borderline personality disorder and focus on findings that may have implications for the treatment of ICD-11 personality disorder severity. We will then present the short-term MBT model, which we have developed for adults [14], and elaborate on how it can be useful and how it may be adapted to children and adolescents. Finally, we offer preliminary suggestions about how this format may or may not fit levels of severity in adolescents.

## 3. MBT for BPD in Adolescence

MBT has gained considerable popularity as a targeted treatment approach for parents, children, adolescents, and families across many levels of personality disorder severity, as evidenced by its wide use in both clinical and non-clinical settings [15,16]. Jørgensen and colleagues [17] recently systematically reviewed the beneficial and harmful effects of psychotherapies for BPD in adolescents. The systematic review identified two randomized clinical trials specifically assessing the effects of MBT for adolescents with self-harm or BPD. In a trial by Rossouw and Fonagy [18] there was evidence of a beneficial effect of MBT compared with treatment as usual in reducing self-harm and depression in adolescents who self-harmed (70% meeting criteria for BPD). The authors found that an increase in mentalizing, a reduction in attachment avoidance, and an improvement in BPD symptoms and traits accounted for the superior effect of MBT, although this evidence must be considered exploratory only. The MBT intervention lasted 12 months and consisted of weekly individual therapy and monthly family therapy. Treatment as usual was not manualized but based on guidance from the UK National Institute for Health. Importantly, the authors reported that there were no statistical differences between treatments in terms of either duration or the use of modalities. The mean duration of the received MBT intervention was approximately 20 h, and approximately one-third of the participants in the MBT intervention did not receive family therapy. Around 50% completed the full 12 months of treatment, which did not differ significantly from the treatment-as-usual group. Finally, in terms of BPD pathology, a large, significant difference emerged at 12 months with only 33% still meeting the criteria for BPD, while the same was true of 58% in the treatment-as-usual group.

Another trial assessing the effects of MBT for adolescents is the M-GAB trial by Beck and colleagues [19], in which 112 adolescents were randomized to 12 months of MBT in a group compared with treatment as usual. The MBT intervention consisted of three MBT introduction sessions, weekly MBT group sessions (slow-open), six MBT-Parent sessions, and five individual case formulation sessions. Treatment as usual consisted of at least 12 individual supportive sessions (not manualized but adapted to the needs of patients, including counseling, psychoeducation, and crisis management). Although positive changes in BPD symptomatology occurred, the results showed no statistical or clinically relevant differences between the MBT intervention and treatment as usual on primary or secondary outcome measures at either end of treatment or follow-up [20]. In terms of BPD psychopathology, only 29% dropped below the cut-off for BPD in both groups and drop-out rates were high: 25% in the treatment-as-usual group and 45% in the MBT group. The authors speculated that group-based MBT may not be suitable for adolescents with high levels of psychopathology and low social functioning and that, following the staging model suggested by Chanen and colleagues [21], group-based intervention may be more appropriate as an early first-stage intervention for adolescents with low levels of severity, possibly corresponding to ICD-11 PD, mild to moderate severity. Subsequent explorative analyses of drop-out and outcomes from this trial found that lower reflective functioning, i.e., mentalizing, predicted drop-out in the MBT intervention [22] and that a more internalizing profile, as opposed to an externalizing one, predicted better outcomes at a two-year follow-up [23]. Overall, these results have led experts in MBT to suggest that more individual and family therapy should be offered to patients with higher personality disorder severity [19], should be of higher intensity and duration [5], and should include a greater focus on and involvement of the social environment (social–ecological approach) [24,25,26].

The effects of MBT have also been assessed in observational studies. In a pilot study by Laurenssen and colleagues [27], 15 inpatients were assessed before and after MBT for adolescents with BPD. The average treatment length was 11 months. The MBT program consisted of four weekly group therapy sessions, one weekly individual session, art therapy, writing therapy, mentalizing cognitive therapy, and a family therapy session every third week. At 12 months’ follow-up, there were significant decreases in symptoms and improved personality functioning. Another small, non-controlled study with 34 patients was performed by Bo and colleagues [28], who reported promising results on several measures of 12 months of MBT group therapy for adolescents with BPD. In addition to weekly group therapy sessions (34 sessions), the adolescents were offered two individual case-formulation sessions and six MBT psychoeducational group sessions, while parents were offered seven sessions of MBT-Parenting, introducing mentalization, personality disorders, attachment, and self-regulation. The authors reported that for 52% of the adolescents, borderline symptoms had dropped below the clinical cut-off at follow-up. The drop-out rate was 26%. However, the results of observational studies should be interpreted with great caution.

## 4. Short-Term MBT for BPD in Adults

MBT currently has empirical support as an 18-month program for adults with BPD [29]. The treatment comprises four essential components: (1) the development of a case formulation at the beginning of treatment, which is continuously reassessed throughout treatment, (2) psychoeducation, (3) weekly group therapy, and (4) weekly individual therapy [11]. While the original 18-month MBT program is the approach prescribed by the MBT manual, this duration is rarely available, and the long and costly treatment combined with a highly prevalent disorder results in insufficient access to evidence-based care.

Hence, a randomized clinical trial is currently underway in which the beneficial and harmful effects of short-term (20-week) compared with long-term (14-month) MBT are being evaluated for adult outpatients with subthreshold or diagnosed BPD [14].

The short-term MBT program is a 20-week psychotherapy course consisting of five sessions of introductory MBT (MBT-I) followed by 15 sessions of group MBT (MBT-G) accompanied by conjoint individual sessions every second week and two psychoeducative meetings with patients and relatives. The groups are closed to enhance cohesion and security among group participants, meaning that all patients start and finish their 20-week program together. Originally, MBT-I was a 12-session introductory psychoeducative program covering relevant topics such as personality disorders, attachment, and mentalization. The original manual has been modified for the five-week intervention [30]. After the completion of MBT-I, the same group of participants will move on to MBT-G, consisting of 15 sessions of MBT in groups, as manualized by Bateman and Fonagy [11]. A copy of our modified manual is available upon request. In our short-term MBT program, group sessions will be accompanied by individual psychotherapy every second week with one of the two group therapists. As part of individual therapy, a case formulation will be prepared and subsequently shared by the participants in the group. The overall purpose of the individual sessions is for the therapist and participant to develop a consensus on the participant’s main difficulties and to establish psychotherapeutic focus points for the group therapy. Patients are furthermore offered three individual follow-up sessions after the end of treatment [7].

## 5. Adapting Short-Term MBT to Adolescents with BPD

The short-term MBT format could potentially have four major advantages for the treatment of adolescents with BPD. First, the use of individual therapy throughout the course of treatment could be an important component for supporting mentalizing, regardless of baseline reflective functioning level. In patients with lower levels of reflective functioning, it may help to prevent drop-out, and in patients with higher levels of reflective functioning, it may help patients to feel continuously supported to share sensitive topics with their individual therapist before sharing with the group. Second, having one of the group therapists double as the individual therapist might enhance the feeling of security, as the adolescent patient will only need to form a therapeutic relationship with two primary therapists throughout the course of treatment, and the individual therapist can help facilitate a safe environment for the patient in the group. Although, we also acknowledge that this format may also be considered a potential disadvantage, as the adolescent may fear that information divulged in individual therapy may somehow be used in group therapy. Third, in contrast to previous MBT formats, the short-term format consists of closed groups, which may facilitate group cohesion and a sense of togetherness and belonging; this latter point may be of particular importance for young patients [24]. Fourth, the short format has the potential to benefit more patients, both from an administrative point of view (more patients will have access to treatment, thus minimizing waitlists) and from a clinical point of view, given that the short timeframe may seem less overwhelming from a youth’s perspective. Five months may seem less of an intrusion into the person’s everyday life compared with the traditional long-term program.

However, considering the immense importance of social environment and family for adolescent development and personality function [31], we believe that further focus on and inclusion of the most important people in the young person’s life will be of the utmost importance. This may simply include offering family therapy, as reported in the Rossouw and Fonagy [18] trial, or it may include one or several network approaches, as described by Bo and colleagues [26] and in the Adolescent Mentalization-Based Integrative Therapy (AMBIT) intervention for the hardest-to-reach adolescents [32]. However, it should also be noted that in the Rossouw and Fonagy trial [18], which arguably has provided the most favorable results, one-third of the patients in the MBT group did not receive the family or network interventions. This could indicate that the importance of providing an individual space for scaffolding and developing mentalizing should not be underrated and that we should not only look at the social system around the adolescents but also more carefully consider the specific psychotherapy processes that are experienced as helpful and foster a more elaborate theory of mind. We suspect that disorganized attachment may have an important role to play in how adolescents can make use of psychotherapy. Recently, Talia and colleagues [33] presented data suggesting that disorganized adult patients display patterns of speech that may hinder the development of epistemic trust in their therapists, which may be an important obstacle to using the therapy process as a vehicle for enhancing a sense of agency and identity. If these results are replicated in adolescents, studying how therapists can intervene would be an important next step in studying the most severe personality-disordered patients described in the ICD-11.

## 6. Translating MBT to the ICD-11 Personality Disorder Model

Hutsebaut and colleagues [5] have presented a very persuasive staging model describing how BPD should not be viewed as a single categorical disorder but instead as a dimensional marker of personality dysfunction disposing the person to a long list of problems, psychopathologies, and psychosocial impairment. Following Hutsebauet and colleagues, we suggest that the short-term MBT program could be used at what the authors label as the “MBT-early” stage of BPD treatment. In categorical terms, this stage is suggested for adolescents aged 13-15 with three to five positive diagnostic BPD criteria. In ICD-11 dimensional terms, this would generally fit the descriptions of mild to moderate personality disorder. Hutsebaut and colleagues suggest that the treatment should focus on individual therapy but also include family sessions, case management, and medication review. In terms of doses, MBT-early consists of an active treatment phase with 16 weekly individual sessions and 3–4 family sessions followed by a booster phase with four booster sessions over 6 months. There are many similarities between this model and the short-term MBT program for adults. However, they differ in two ways: First, the exclusion of MBT group therapy as a modality, which is only suggested at the most severe level in the model proposed by Hutsebaut and colleagues. Secondly, they differ in the lack of structured psychoeducation, which is suggested mainly as a preventive, subclinical intervention in Hutsebaat and colleagues’ MBT-early program. Decisions about the inclusion or exclusion of modalities such as group therapy and psychoeducation at different levels of ICD-11 severity is ultimately an empirical question. However, from a clinical perspective, including both group therapy and psychoeducation seems very justified, perhaps especially at the mild–moderate severity level. First, group therapy may provide exactly the kind of training arena for developing the mentalizing skills that are needed for disturbances at this level of ICD-11 PD. For example, the ICD-11 states that “relationships may be characterized by dependence and avoidance of conflict by giving in to others, even at some cost to themselves” [2] (p. 379). In the MBT group, this problem would in all probability come to the surface and could be mentalized both in the group and individual therapy. In the short-term MBT program [7], this work is highly facilitated by the fact the individual therapist is also one of the group therapists. Thus, instead of relying solely on the individual young patient’s account of interpersonal events, the therapist may support mentalizing by probing different situations in which he or she felt that something important was going on with the person. In many ways, this seems to mimic what an active, engaged, and mentalizing parent does in a family [34]. Second, although the evidence for psychoeducation as an intervention modality is not strong for either adults or adolescents [35], psychoeducation does play a very important role in the general MBT model. It is often described as an initial component in developing epistemic trust, as it involves the therapist providing information or a model of the mind that matches something in the patient’s experience and often furthers their understanding of themselves [36]. This may happen through something as simple as providing labels for experiences—for example, that a particular experience is called a “developmental trauma”, or explaining that problems with understanding other people may be partly attributed to not always being well-understood by those around you. Furthermore, psychoeducation may be viewed as an example of not just how the mind works, but, from a more general mentalizing perspective, of the development of epistemic trust—how personally and culturally useful information is exchanged from mind to mind [6]. We would argue that psychoeducational information is not just useful in itself—as a modality of therapy, it also, and more importantly, provides a key context for identifying and eventually alleviating problems with epistemic trust.

A major future challenge in matching ICD-11 personality disorder severity with optimal treatments along the full spectrum of disorder is finding valid and reliable ways to demarcate the different levels. This may be even more challenging in younger people, simply due to their shorter life experience. Consider some of the specific examples of severe personality disorder in ICD-11: “The individual is largely unable to set and pursue realistic goals” or “The individual is unwilling or unable to sustain regular work due to lack of interest or effort, poor performance (e.g., failure to complete assignments or perform expected roles, unreliability), interpersonal difficulties, or inappropriate behaviour (e.g., fits of temper, insubordination)” [2] (p. 381). After how many years is it reasonable to assess that such problems are clearly present? At what point should such problems primarily be reduced to a personality problem, as opposed to a contextual or even a societal problem? Clinicians quite rightly hesitate to assign a personality disorder diagnosis to adolescents, but this reluctance may also prevent adolescents from accessing potentially effective treatments at the time when the treatment is most effective [37]. The ICD-11 personality disorder severity descriptions have come a long way in describing personality pathology in a more psychological and non-stigmatizing language, but, as shown by the examples above, certain examples may still be difficult for clinicians to assess, especially in child and adolescent populations. In these cases, and especially in assigning an ICD-11 personality disorder diagnosis of severe degree, the general note used throughout the PD diagnostic guidelines specifying that “the list of examples is not exhaustive and not intended to suggest that all items will be present in any single individual” [2] (p. 381) seems extremely useful. In severe cases, clinicians may focus more on psychological and distress descriptors, which may ultimately help to channel those in need of evidence-supported treatments such as MBT.

## 7. Conclusions and Future Directions

The short-term MBT program, originally developed to treat adult BPD, can be a potentially effective psychological intervention when adapted to adolescents with ICD-11 personality disorder. Short-term MBT includes individual therapy, uses a combined format in which the group therapist is also the individual therapist, is run in closed groups where patients start and finish the group together, and is shorter than existing psychotherapy options for adolescents with personality disorders. Together, we speculate that these factors could benefit an adolescent population, as they enhance a feeling of security and cohesion, support mentalizing in a safe environment, and are less overwhelming from a youth’s perspective. We speculate that this adaptation could potentially prevent drop-out and enhance outcomes for adolescents with ICD-11 personality disorder. Developing efficient short-term treatment for a diagnosis that traditionally has been suggested to require treatment of a longer duration could potentially have a large impact on public health policies and guidelines.

A recent systematic review assessing the effects of psychotherapies for adolescents with BPD [17] concluded that it is currently difficult to conclude whether psychotherapy, in general, is effective for adolescents with BPD, due to the high risk of bias, high attrition rates, and small trials underpowered to confirm or reject realistic intervention effects on patient-important outcomes. Therefore, there is a need for more high-quality trials with larger samples to identify potentially effective psychological interventions for adolescents with BPD. As we have outlined in this paper, the short-term MBT program can be a promising intervention for adolescents with ICD-11 personality disorder. To test this assumption, we suggest that the next step be a large, randomized clinical trial randomizing adolescents into an adapted short-term MBT program, as outlined in this paper, or a control intervention using a psychological sham intervention. Outcomes would be selected based on what is most important to patients (e.g., symptoms, quality of life, level of functioning) but would also include a comprehensive assessment of harmful effects, including objective outcomes such as suicide, suicide attempts, self-harm, and serious adverse events, and the trial should be adequately powered to assess these outcomes. That way, we could move closer to potentially establishing short-term MBT as an evidence-based intervention for adolescents with personality disorders, which would significantly benefit the care of individual patients in the future. We acknowledge that a major limitation of the ideas presented in this conceptual paper is that the empirical literature was not systematically reviewed. The reader should take this into account when assessing the potential benefits and harms of the ideas presented.

## Figures and Tables

**Table 1:** A new psychological language for personality disorders: Crosswalk examples between ICD-10 diagnostic categorical criteria and potential aspects determining ICD-11 severity.

ICD-10	ICD-11
Tendency to bear grudges persistently, e.g., refusal to forgive insults and injuries or slights (Paranoid PD).	Interpersonal dysfunction (ability to manage conflict in relationships).
Apparent indifference to either praise or criticism (Schizoid PD).	Emotional manifestation (tendency to be emotionally underreactive).
Incapacity to experience guilt or to profit from experience, particularly punishment (Dissocial PD).	Emotional manifestation (range and appropriateness of emotional experience and expression).
Excessive efforts to avoid abandonment (BPD).	Interpersonal dysfunction (ability to develop and maintain close and mutually satisfying relationships).
Shallow and labile affectivity (Histrionic PD).	Emotional manifestations (range and appropriateness of emotional experience).
Perfectionism that interferes with task completion (Anankastic PD).	Cognitive manifestations (appropriate stability and flexibility of belief systems).
Excessive preoccupation with being criticized or rejected in social situations (Avoidant PD).	Interpersonal dysfunction (ability to understand and appreciate others’ perspectives).
Limited capacity to make everyday decisions without an excessive amount of advice and reassurance from others (Dependent PD).	Self-dysfunction (capacity for self-direction).

Notes. Abbreviations: PD = personality disorder; BPD = borderline personality disorder.

## Data Availability

No new data were created or analyzed in this study. Data sharing is not applicable to this article.
